# An evaluation of Birmingham Own Health^® ^telephone care management service among patients with poorly controlled diabetes. a retrospective comparison with the General Practice Research Database

**DOI:** 10.1186/1471-2458-11-707

**Published:** 2011-09-19

**Authors:** Rachel E Jordan, Robert J Lancashire, Peymané Adab

**Affiliations:** 1 Unit of Public Health, Epidemiology & Biostatistics, Public Health Building, University of Birmingham, Edgbaston, Birmingham, UK

## Abstract

**Background:**

Telephone-based care management programmes have been shown to improve health outcomes in some chronic diseases. Birmingham Own Health^® ^is a telephone-based care service (nurse-delivered motivational coaching and support for self-management and lifestyle change) for patients with poorly controlled diabetes, delivered in Birmingham, UK. We used a novel method to evaluate its effectiveness in a real-life setting.

**Methods:**

Retrospective cohort study in the UK. 473 patients aged ≥ 18 years with diabetes enrolled onto Birmingham Own Health^® ^(intervention cohort) and with > 90 days follow-up, were each matched by age and sex to up to 50 patients with diabetes registered with the General Practice Research Database (GPRD) to create a pool of 21,052 controls (control cohort). Controls were further selected from the main control cohort, matching as close as possible to the cases for baseline test levels, followed by as close as possible length of follow-up (within +/-30 days limits) and within +/-90 days baseline test date. The aim was to identify a control group with as similar distribution of prognostic factors to the cases as possible. Effect sizes were computed using linear regression analysis adjusting for age, sex, deprivation quintile, length of follow-up and baseline test levels.

**Results:**

After adjusting for baseline values and other potential confounders, the intervention showed significant mean reductions among people with diabetes of 0.3% (95%CI 0.1, 0.4%) in HbA1c; 3.5 mmHg (1.5, 5.5) in systolic blood pressure, 1.6 mmHg (0.4, 2.7) in diastolic blood pressure and 0.7 unit reduction (0.3, 1.0) in BMI, over a mean follow-up of around 10 months. Only small effects were seen on average on serum cholesterol levels (0.1 mmol/l reduction (0.1, 0.2)). More marked effects were seen for each clinical outcome among patients with worse baseline levels.

**Conclusions:**

Despite the limitations of the study design, the results are consistent with the Birmingham Own Health^® ^telephone care management intervention being effective in reducing HbA1c levels, blood pressure and BMI in people with diabetes, to a degree comparable with randomised controlled trials of similar interventions and clinically important. The effects appear to be greater in patients with poorer baseline levels and the intervention is effective in the most deprived populations.

## Background

Increasing numbers of people worldwide are living with a long-term condition such as diabetes, heart failure and heart disease. Poorly controlled chronic conditions can lead to more rapid deterioration, complications, poor quality of life and intensive use of the health services. The emphasis of care in the UK has now changed from being predominantly reactive to a more preventive management approach [1], with the aim of reducing emergency hospital admissions and encouraging patients to make healthier choices about diet, physical activity and lifestyle through self-management of their condition [2].

Approximately 2.7% of adults aged 20-64 years in England have diagnosed diabetes [3]. People with diabetes have a high risk of cardiovascular mortality and therefore control of cardiovascular risk factors is essential to prevention. Although the primary care management of diabetes has changed to meet newer guidelines and control of blood pressure and cholesterol have improved, glycaemic control and obesity levels have not [4,5].

Systematic reviews have shown that some interventions may produce improvements in disease management [6-12], but in general, further research is required as to the best method of delivery to attain sustained improvements in glucose control and other outcomes.

Care management programmes using the telephone have been shown in other chronic diseases, such as heart failure and coronary heart disease, to improve some health outcomes and also reduce health service use [13-15]. They offer an attractive easy-access, inexpensive approach. There are several published studies of telephone care management of people with diabetes, although few good quality RCTs [9]. A cluster RCT in the US [16] (IDEATel) of 1665 Medicare recipients aged 55 years and older with diabetes showed that in the intervention group (nurse case manager, video conferencing, glucose and BP monitoring, educational website and some coaching/motivational aspect) at 1 year, there were significant improvements in HbA1c (0.18% p = 0.0006), SBP, DBP, total and LDL cholesterol compared with the control group. An RCT in Salford of Pro-Active Call Centre Treatment Support (PACCTS) in 591 patients with type 2 diabetes (patient education, metabolic management, motivational techniques, customised frequency of contacts) showed a significant improvement in HbA1c compared with controls at 1 year. However, the effect was mainly seen in patients with > 7% HbA1c at baseline, with a relative improvement of 0.49% [17].

Birmingham Own Health^® ^is a bespoke telephone-based care management service for patients with poorly-controlled diabetes, based in Birmingham East and North Primary Care Trust, and led by nurses who provide motivational coaching and support for self-management and lifestyle changes within a personalized care plan. In this resource-scarce environment, it is essential that new services are properly evaluated. As the service was already in place, it was not possible to undertake a randomised controlled trial. In order to compare its effectiveness against usual care, we used the General Practice Research Database (GPRD) in a unique way, obtaining a matched cohort of patients and comparing a range of clinical indicator outcomes.

## Methods

### Study design

Retrospective cohort study comparing Birmingham Own Health^® ^patients, receiving their personalised telephone care intervention, with a matched control cohort from the General Practice Research Database (GPRD), receiving usual care.

### Intervention cohort

#### Description of Birmingham Own Health^® ^intervention

Birmingham Own Health^® ^is a "bespoke disease management programme which supports individuals with long-term conditions to make changes in their daily behaviour that will have a positive impact on their health and will encourage more appropriate use of NHS services" [18]. It is a partnership between Birmingham East and North PCT, Pfizer Health Solutions and NHS Direct, commissioned in 2005 to deliver care management support by telephone to patients with diabetes, cardiovascular disease and heart failure. This paper refers to patients with diabetes. Patients are recommended by their GP according to need; those with poorly controlled conditions are referred and contacted directly, although they may "opt-out" when approached. Each consenting individual is assigned a care manager (specifically trained nurses employed by NHS Direct), and together they develop a personalized care plan with support from decision-support software. Care managers make pro-active telephone calls and provide motivational coaching with stage-based counselling and support for self-management (including adherence to medication and treatment goals) and lifestyle change (such as diet and exercise). Care managers follow five fundamental steps: assessment, recommendation, follow-up, ongoing monitoring and evaluation. Several key recommendations were used to underpin the decision-support software tool, developed from evidence-based literature and consensus guidelines, including that of the Joint British Societies 2 (2005) [19], and National Service Framework for Diabetes [20]. The target for HbA1c was < 6.5%; for blood pressure < 130/80; total cholesterol < 4.0 mmol/L (or ≥ 25% reduction); BMI 18.5-24.9.

The nurses receive a six-week training process which includes all aspects of care-management and education related to diabetes, and in the specific delivery of the service. Although there is a broad protocol covering the 8 self-management priorities (see below), they are trained to provide telephone-based advice which is completely individual and tailored to each person's condition and circumstances, providing supportive not directive care management, allowing each individual to set their own goals. Nurses have a robust clinical background and are trained to work with the primary care team where needed.

Birmingham Own Health^® ^eight self-management priorities:

1. Know how and when to call for help

2. Learn about the condition and set goals

3. Take medicines correctly

4. Get recommended tests and services

5. Act to keep the condition well-controlled

6. Make lifestyle changes and reduce risks

7. Build on strengths and overcome obstacles

8. Follow-up with specialists and appointments

Care managers are able to discuss with the healthcare staff from the patient's general practice to help understand their patient's condition and can also make referrals for review. Participants also receive educational materials and a hand-held record book. They remain in the programme for nine months, with 1-2 calls per month of varying length (according to need). At this stage, they undertake a care-review where they may "graduate" or remain in the programme. It is based on the "Green Ribbon" model designed in the US [21]. The programme began recruiting on 1/4/2006 and is ongoing.

#### Population receiving Birmingham Own Health^® ^intervention

Patients were eligible for the Birmingham Own Health^® ^diabetes module if they were aged 18 years and over, were on the diabetes mellitus register, had access to a telephone, and at least one of the following:

• Cholesterol ≥ 5 mmol/l (due to non-compliance with their medication)

• CHD or Stroke/TIA registry

• Hypertension registry or BP > 150/90

• Complication of retinopathy, micro or overt proteinuria, lower extremity amputation or ulcer, diabetic neuropathy

• Assessed by clinician as likely to benefit from the service (because of poor compliance)

Patients with diabetes were also included if they had heart failure or cardiovascular disease and had been referred to Birmingham Own Health^® ^for those conditions. Inclusion was not dependent on medications received.

#### Population included in these analyses

This is an ongoing service and therefore participants had experienced differing amounts of time in the programme. In order to give reasonable time for some effect, participants were only included in the analyses for this study if they had ≥ 90 days follow-up data for the outcome of interest. This sub-group had many characteristics similar to that of the whole Birmingham Own Health group except for smoking status where information was more complete, and also there were higher reported levels of depression, and use of beta-blockers, diuretics, statins and insulin. The first recorded data for each outcome was classified as the baseline data, and the latest recorded data before 31/12/2007 as their final data. Any data before 1/4/2006 was carried forward to 1/4/2006 as this was when the intervention commenced.

#### Descriptive data obtained

Information on demographic details, smoking history, other medical conditions and medications were obtained by self-report. Baseline clinical data were obtained directly from the GPs, although outcome data were obtained by self-report, where patients took a hand-held booklet to their GP to record measurements and tests (this should reflect confidential GP records). A measure of deprivation (Index of Multiple Deprivation 2004) was obtained for the ward of the GP practice of the patient. Patients with missing deprivation scores were assigned quintile 5 as the intervention was directed towards practices in the most deprived areas.

### Control cohort

#### General Practice Research Database (GPRD)

Controls were obtained from the GPRD. The GPRD is a computerised database of anonymised longitudinal medical records from about 488 general practices (over 3.6 million existing patients) throughout the UK, including patient demographics, medical diagnoses, prescription information, referral and treatment outcomes [22]. The population included in the database is broadly representative of the demographic breakdown of the UK population. The data has been used for cross-sectional, case-control and cohort analyses. Over 1.4 million patients in the database have more than 11 years data available.

### Selection of control cohort

#### Phase I: Selection of a large pool of controls

Patients were selected from the GPRD for the control cohort if they met the following criteria:

• Registered with general practices in England contributing to the GPRD at an "up-to-standard" quality level from two months prior to 1/4/2005 (excluding practices involved in Birmingham Own Health^® ^Intervention)

• Aged 18 years or over

• Diagnosed with diabetes mellitus before 1/04/2005

• Alive and with complete follow-up at 31/12/2007.

It was not possible to identify patients with poor disease control from the GPRD data.

Exclusion criteria were derived from that used for the Birmingham Own Health^® ^Intervention and therefore the following were excluded:

• Patients with a diagnosis of dementia in the last 5 years

• Patients with diagnosis of schizophrenia or manic depression or a prescription for treatment with anti-psychotics in the last 5 years

• Patients with a record of drug addiction or a prescription record for methadone in the last 5 years

• Women with a record of pregnancy in the last 5 years

• Patients with a terminal condition such as Parkinson's Disease, motorneurone disease, Huntingdon's chorea, or any malignant cancer in the last 5 years

• Patients with renal dialysis or prescription of anti-retroviral therapy (to indicate HIV) or HIV diagnosis in the last 5 years

**Figure 1:**
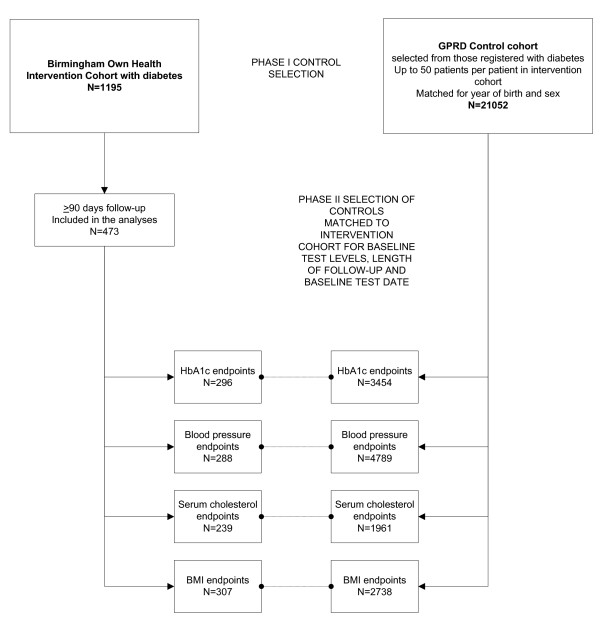
**Flow chart indicating matching and selection of controls from the GPRD**.

The process of selecting matched controls is illustrated in Figure 1. Up to 50 GPRD patients per patient in the intervention cohort were selected to form the control pool, matched on year of birth and sex. There were insufficient GPRD patients with diabetes to also match on deprivation quintile. Codelists used to search the GPRD for each condition were selected in collaboration with the GPRD and were designed to be inclusive (these are available from the authors on request).

#### Phase II: further selection of controls for each outcome

Each outcome was evaluated separately. Controls were further selected from the main pool, matching as close as possible to the cases for baseline test levels, followed by as close as possible length of follow-up (within +/-30 days limits) and within +/-90 days baseline test date. Duplicate controls were removed so that each control contributed only once to each outcome. There was no limit to the number of controls selected per case. The aim was to identify a control group with as similar distribution of prognostic factors to the cases as possible.

#### Descriptive information extracted from the GPRD

Information on demographic details, smoking history, other medical conditions and medications was extracted. Smoking status within the GPRD was classified as latest reported before 1/4/2006. However in order to reduce misclassification of reported never smoking controls from the GPRD, prior records were searched to capture "ever smokers". Medication use within the GPRD was that recorded in the two years prior to 1/4/2006.

### Outcomes

The primary outcomes were glycosylated haemoglobin (HbA1c), systolic (SBP) and diastolic blood pressure (DBP), total serum cholesterol and Body Mass Index (BMI).

### Statistical analyses

The effect of the intervention on the primary outcomes was evaluated using least-squares linear regression of final values, presenting mean differences (with 95% confidence intervals) adjusted for age, sex, smoking history, and baseline levels of blood pressure, HbA1c, cholesterol, BMI (as appropriate for that outcome) (ie an ANCOVA approach). Sub-group analyses were conducted according to age, sex, deprivation quintile, baseline test levels and length of follow-up.

### Ethical approval

Ethical approval was obtained from the University of Birmingham ethics committee and approval to use GPRD data was obtained from the Independent Scientific Advisory Committee (ISAC). The chairman of Birmingham East and North Local Research Ethics Committee approved the use of the Birmingham Own Health^® ^data as a service evaluation.

## Results

### Characteristics of patients

Characteristics of both the intervention and control patients are given in Table 1. Of a total of 1195 participants enrolled on the intervention, 473 had at least 90 days follow-up for one or more of the primary outcomes.

**Table 1:** Characteristics of included patients

Characteristic	Birmingham Own Health **Intervention**^® ^**(included in** analyses)	GPRD comparison (usual care)
**N**	473	21,052

**Mean age (SD)**	62.4 (13.0)	62.8 (12.7)

**Sex**		
Male	263 (55.6%)	11,820 (56.2%)
Female	210 (44.4%)	9232 (43.9%)

**Smoking status**		
Never smoker	175 (37.0%)	8661 (41.4%)
Ex-smoker	157 (33.2%)	9134 (43.4%)
Current smoker	75 (15.9%)	3144 (14.9%)
Unknown	66 (14.0%)	113 (0.5%)

**Deprivation quintile of practice**		
1 (least deprived)	9 (1.9%)	3479 (16.5%)
2	0	2532 (12.0%)
3	0	4401 (20.9%)
4	90 (19.0%)	4174 (19.8%)
5 (most deprived)	374 (79.1%)	6466 (30.7%)

**Other conditions**		
Coronary Heart Disease	160 (33.8%)	3646 (17.3%)
Congestive Heart Failure	15 (3.2%)	470 (2.2%)
Asthma	36 (7.6%)	2948 (14.0%)
Depression	97 (20.5%)	3545 (16.8%)
Stroke	24 (5.1%)	848 (4.0%)
COPD	9 (1.9%)	695 (3.3%)

**Medications**		
Betablockers	123 (26.0%)	5217 (24.8%)
Thiazides*	178 (37.6%)	5769 (27.4%)
Statins	333 (70.4%)	15,202 (72.2%)
Digoxin/digitoxin	18 (3.8%)	437 (2.1%)
Insulin	170 (35.9%)	5505 (26.2%)

* For the Birmingham Own Health^® ^Intervention this includes all diuretics

The 473 included participants were compared to controls taken from a pool of 21,052 matched control patients identified from the GPRD. The intervention group had a similar age and sex profile to the control group, although had a greater number of current smokers and a different deprivation profile. Although many of the comorbidities and medications had similar rates between the two cohorts, those in the Birmingham Own Health cohort reported lower rates of asthma and COPD, although higher rates of depression, use of diuretics and insulin.

In addition, there was a greater proportion of participants in Birmingham Own Health^® ^with heart disease (37.0% were also enrolled on the CHD or heart failure modules) compared with those identified as having heart disease from the GPRD (19.9%).

### Effect of Birmingham Own Health^® ^intervention among people with diabetes

Table 2, Table 3, Table 4 and Table 5 indicate the effect of the Birmingham Own Health^® ^Intervention on people with diabetes, for each of the primary outcomes. The average length of follow-up ranged between 296 days (8 months) and 336 days (11 months) depending on the outcome. For all outcomes except serum cholesterol, patients in the intervention group had slightly higher baseline values than those in the control group. Patients receiving the intervention showed reductions for each of the outcomes over the follow-up period, although patients in the control group remained relatively stable over time in comparison. After adjusting for baseline values and other potential confounders, the intervention showed significant mean reductions of 0.3% (95%CI 0.1, 0.4%) in HbA1c; 3.5 mmHg (1.5, 5.5) in systolic blood pressure and 1.6 mmHg (0.4, 2.7) in diastolic blood pressure, and 0.7 kg/m^2 ^reduction in BMI (0.3, 1.0). The effect on serum cholesterol (reduction of 0.1 mmol/l (0.1, 0.2)) was relatively smaller but remained significant. For each of the outcomes, poorer baseline values tended to lead to larger reductions, with patients with ≥ 8.0% HbA1c at baseline experiencing 0.4% improvement over the period of the study, those with SBP of ≥ 150 a reduction of 5.4 mmHg, and those with high BMI of ≥ 40 a 2.9 point reduction.

**Table 2:** Effect of Birmingham Own Health^® ^Intervention on HbA1c values among people with diabetes

	Intervention	Control	Multivariate linear regression coefficients (95%CI)
N	296	3454	

Mean age (years) (SD)	61.6 (13.1)	63.5 (11.6)	-0.007 (-0.01, -0.004)

Males (n (%))	155 (52.4%)	1828 (52.9%)	-0.03 (-0.10, 0.04)

Smoking status			
Never smoker	118 (39.9)	1403 (40.6%)	1.0
Ex-smoker	88 (29.7)	1601 (46.4%)	0.008 (-0.07, 0.08)
Current smoker	42 (14.2%)	450 (13.0%)	0.02 (-0.08, 0.13)
Unknown	48 (16.2%)	-	

Length of follow-up (days)			
Mean (SD)	331.9 (136.2)	336.3 (132.9)	0.0002 (-0.0001,
Median (IQR)	328 (216-449)	345 (219-441)	0.0004)

Baseline HbA1c (%) Mean (SD)	8.2 (1.8)	7.7 (1.5)	0.70 (0.68, 0.73)
*Sub-groups (n (%))*			
*< 7.0%*	*73 (24.7%)*	*1156 (33.5%)*	
*7.0-7.9%*	*86 (29.1%)*	*1106 (32.0%)*	
*8.0-8.9%*	*59 (19.9%)*	*620 (18.0%)*	
*> 9.0%*	*78 (26.4%)*	*572 (16.6%)*	

Final value (mean) (SD)	7.8 (1.6)	7.7 (1.5)	

Mean change (SD)	-0.4 (1.4)	-0.03 (1.1)	

Difference in final values (intervention - control) (95% CI)	+0.11 (-0.07, 0.29)		

Adjusted mean difference in final HbA1c values between intervention and control (%) (95%CI)*			**-0.25 (-0.38, -0.11)**

Adjusted mean difference in final HbA1c values between intervention and control by sub-group of baseline values (95%CI) (%)			
< 7.0%			0.2 (-0.01, 0.4)
7.0-7.9%			-0.3 (-0.6, -0.1)
8.0-8.9%			-0.3 (-0.6, 0.1)
> 9.0%			-0.6 (-1.0, -0.2)

* adjusted for age, sex, baseline values, smoking status, length of follow-up

**Table 3:** Effect of Birmingham Own Health^® ^Intervention on diastolic and systolic blood pressure among people with diabetes

	Intervention	Control	Multivariate linear regression coefficients (95%CI)	
			**Diastolic blood pressure**	**Systolic blood pressure**

N	288	4789		

Mean age (years) (SD)	63.3 (12.4)	65.3 (11.2)	-0.13 (-0.15, -0.11)	0.12 (0.08, 0.16)

Males (n (%))	155 (53.8%)	2532 (52.9%)	0.26 (-0.26, 0.79)	-0.12 (-1.02, 0.78)

Smoking status				
Never smoker	119 (41.3%)	1963 (41.0%)	1.0	1.0
Ex-smoker	99 (34.4%)	2256 (47.1%	-0.55 (-1.11, 0.006)	-0.07 (-1.03, 0.89)
Current smoker	34 (11.8%)	570 (11.9%)	-1.36 (-2.20, -0.51)	0 (-1.44, 1.44)
Unknown	36 (12.5%)	-		

Length of follow-up (days)				
Mean (SD)	321.6 (143.4)	325.7 (139.5)	-0.0006 (-0.002, 0.001)	0.001 (-0.002, 0.004)
Median (IQR)	321 (184, 445.5)	338 (198, 440)		

Baseline diastolic blood pressure (mmHg) Mean (SD)	8.2 (1.8)	7.7 (1.5)	0.38 (0.36, 0.41)	-
*Sub-groups (n (%))*				
*< 80 mmHg*	*145 (50.3%)*	*2645 (55.2%)*		
*80-89 mmHg*	*107 (37.2%)*	*1688 (35.2%)*		
*90-99 mmHg*	*24(8.3%)*	*385 (8.0%)*		
*> 100 mmHg*	*12 (4.2%)*	*71 (1.5%)*		

Baseline systolic blood pressure (mmHg) Mean (SD)	139.6 (18.0)	138.3 (14.6)	-	0.39 (0.36, 0.42)
*Sub-groups (n (%))*				
*< 120 mmHg*	*27 (9.4%)*	*341 (7.1%)*		
*120-129 mmHg*	*48 (16.7%)*	*755 (15.8%)*		
*130-139 mmHg*	*80 (27.8%)*	*1346 (28.1%)*		
*140-149 mmHg*	*71 (24.7%)*	*1389(29.0%)*		
*> 150 mmHg*	*62 (21.5%)*	*958 (20.0%)*		
				

Final value (mean) (SD) (mmHg)				
Diastolic blood pressure	75.2 (9.3)	76.1 (10.2)		
Systolic blood pressure	134.6 (15.9)	137.6 (16.8)		

Mean change (SD) (mmHg)				
Diastolic blood pressure	-3.3 (11.7)	-0.6 (10.8)		
Systolic blood pressure	-5.0 (19.0)	-0.7 (17.8)		

Difference in final values (mmHg)				
(intervention - control) (95% CI)				
Diastolic blood pressure	-0.9 (-2.1, 0.3)			
Systolic blood pressure	-3.0 (-5.0, -1.0)			

**Adjusted mean difference in final blood pressure** between intervention and control* (mmHg) (95%CI)			**-1.6 (-2.7, -0.4)**	**-3.5 (-5.5, -1.5)**

Adjusted mean difference in final blood pressure between intervention and control by sub-group of baseline values (95%CI) (mmHg)			< 80 mmHg -1.0 (-2.6, 0.6) 80-89 mmHg -1.6 (-3.5, 0.3) 90-99 mmHg -3.1 (-7.7, 1.5) ≥ 100 mmHg -0.1 (-8.4, 8.2)	< 120 mmHg -1.9 (-8.1, 4.4) 120-129 mmHg -2.7 (-7.4, 1.9) 130-139 mmHg -1.6 (-5.2, 2.0) 140-149 mmHg -4.6 (-8.5, -0.8) ≥ 150 mmHg -5.4 (-10.4, -0.4)

* adjusted for age, sex, baseline values, smoking status, length of follow-up

**Table 4:** Effect of Birmingham Own Health^® ^Intervention on serum cholesterol among people with diabetes

	Intervention	Control	Multivariate linear regression coefficients (95%CI)
N	239	1961	

Mean age (years) (SD)	63.1 (11.7)	64.4 (10.9)	-0.001 (-0.005, 0.002)

Males (n (%))	129 (54.0)	1024 (52.2)	-0.15 (-0.22, -0.08)

Smoking status			
Never smoker	87 (36.4%)	824 (42.0)	1.0
Ex-smoker	92 (38.5%)	906 (46.2)	-0.03 (-0.11, 0.04)
Current smoker	29 (12.1%	231 (11.8)	0.01 (-0.10, 0.12)
Unknown	31 (13.0%)	-	

Length of follow-up (days)			
Mean (SD)	322.7 (134.4)	330.4 (122.9)	-0.0001 (-0.0004, 0.0002)
Median (IQR)	313 (215, 419)	343 (235, 405)	

Baseline serum cholesterol (mmol/l) Mean (SD)	4.3 (1.0)	4.4 (1.0)	0.60 (0.56, 0.63)
*Sub-groups (n (%))*			
*< 4 mmol/l*	*88 (36.8%)*	*656 (33.5%)*	
*4.0-4.9 mmol/l*	*82 (34.3%)*	*787 (40.1%)*	
*5.0-5.9 mmol/l*	*46 (19.2%)*	*310 (15.8%)*	
*6.0-6.9 mmol/l*	*9 (3.8%)*	*75 (3.8%)*	
*> 7.0 mmol/l*	*3 (1.3%)*	*33 (1.7%)*	

Final serum cholesterol (mmol/l) (mean) (SD)	4.1 (1.1)	4.3 (1.0)	

Mean change in serum cholesterol (mmol/l) (SD)	-0.2 (1.0)	-0.1 (0.9)	

Difference in final serum cholesterol (intervention - control) (95% CI)	-0.2 (-0.3, -0.0)		

Adjusted mean difference in final serum cholesterol between intervention and control (mmol/l)(95%CI)*			**-0.1 (-0.2, -0.1)**

Adjusted mean difference in final serum cholesterol between intervention and control by sub-group of baseline values (mmol/l) (95%CI)			
*< 4 mmol/l*			0.0 (-0.2, 0.2)
*4.0-4.9 mmol/l*			-0.3 (-0.5, -0.1)
*5.0-5.9 mmol/l*			-0.0 (-0.3, -0.3)
*6.0-6.9 mmol/l*			-0.1 (-0.9, 0.7)
*> 7.0 mmol/l*			-0.7 (-2.6, 1.2)

* adjusted for age, sex, baseline values, smoking status, length of follow-up

**Table 5:** Effect of Birmingham Own Health^® ^Intervention on BMI among people with diabetes

	Intervention	Control	Multivariate linear regression coefficients (95%CI)
N	307	2738	

Mean age (years) (SD)	63.1 (12.3)	64.0 (11.1)	-0.03 (-0.04, -0.02)

Males (n (%))	166 (54.1)	1457 (53.2)	-0.15 (-0.38, 0.07)

Smoking status			
Never smoker	111 (36.2%)	1130 (41.3%)	1.0
Ex-smoker	108 (35.2%)	1274 (46.5%)	0.23 (-0.01, 0.47)
Current smoker	44 (14.3%)	334 (12.2%)	-0.23 (-0.59, 0.11)
Unknown	44 (14.3%)	-	

Length of follow-up (days)			
Mean (SD)	296.2 (139.1)	307.3 (131.4)	0.0002 (-0.0007, 0.001)
Median (IQR)	314 (191, 390)	298 (173, 390)	

Baseline BMI (kg/m^2^) Mean (SD)	31.2 (7.1)	30.9 (7.3)	0.90 (0.88, 0.91)
*Sub-groups (n (%))*			
*< 25*	*49 (16.0%)*	*424 (15.5%)*	
*25-29*	*97 (31.6%)*	*938 (34.3%)*	
*30-34*	*85 (27.7%)*	*765 (27.9%)*	
*35-39*	*50 (16.3%)*	*360 (13.1%)*	
*> 40*	*26 (8.5%)*	*251 (9.2%)*	

Final BMI (kg/m^2^) (mean) (SD)	30.7 (6.4)	31.1 (7.3)	

Mean change in BMI (kg/m^2^) (SD)	-0.5 (3.6)	0.2 (3.0)	

Difference in final BMI (kg/m^2^) (intervention - control) (95% CI)	-0.4 (-1.3, 0.4)		

Adjusted mean difference in final BMI between intervention and control (kg/m^2^) (95%CI)*			**-0.7 (-1.0, -0.3)**

Adjusted mean difference in final BMI between intervention and control by sub-group of baseline values (kg/m^2^) (95%CI)			
< 25			0.5 (-0.2, 1.2)
25-29			-0.5 (-0.8, -0.2)
30-34			-0.7 (-1.4. 0.0)
35-39			-1.4 (-2.1, -0.6)
≥ 40			-2.9 (-5.7, -0.1)

* adjusted for age, sex, baseline values, smoking status, length of follow-up

### Sub-group analyses

Repeating the analyses restricting to participants from the two most deprived quintiles had little effect on the results (data not shown). Females had a tendency to achieve greater improvements in blood pressure (5.1/2.5 mmHg SBP/DBP compared with 2.1/0.8 in males), and a possible greater reduction in BMI (1.4 among females, compared with 0.1 points among males). Other than the reduction in blood pressure, where the intervention may have had greater immediate effects (3-6 mths) than in the longer term, there was no consistent effect of length of follow-up. The reduction in HbA1c was more marked among those aged 65 years and over (0.4% (95%CI 0.2, 0.6%) than among those younger than 65 years (0.2% (95%CI 0.0, 0.3%)).

## Discussion

As far as we are aware, the use of the GPRD to obtain controls is a novel way to evaluate public health interventions. We found that participants with diabetes in the Birmingham Own Health^® ^intervention had both statistically and clinically significantly lower HbA1c and blood pressure and BMI over an average 10 months follow up, compared to matched controls from the GPRD. The overall effect on total cholesterol was slightly lower, but still statistically significant. Furthermore, in line with other studies [17], those with poorest levels of control at baseline showed the highest level of improvement, again compared to matched controls.

The magnitude of effect for some of the clinical end-points was similar to that seen in randomised controlled trials of similar interventions. Although also including different elements (eg videoconferencing, telemonitoring) the IDEATel trial of 1,665 people with diabetes identified through disadvantaged primary care practices in New York, found very similar reductions in HbA1c (-0.18%), SBP (-3.4 mmHg) and DBP (-1.9 mmHg) at one year [16], to those seen in our study among intervention, compared to control participants. A recent follow-up study found that the benefits in terms of HbA1c, cholesterol and blood pressure were maintained after 5 years, although there were no differences in mortality rates between control and intervention groups [11]. Similarly the PACCTS trial (telephone care management in Salford) comprising 591 people with diabetes from primary care, showed similar results, with reductions in HbA1c of -0.31% (and -0.49% in those with HbA1C ≥ 7.0% at baseline) in the intervention compared to the usual care arm [17]. Systematic reviews of self-management interventions have shown that mean reductions in HbA1c range from 0.26% [23], 0.45% [24] to 0.81% [25]. The reviews suggest that intervention effects reduce over time [23], and that regular re-enforcement during follow-up and interventions that involve patient collaboration are more likely to succeed [23]. Cardiovascular outcomes including blood pressure change are less frequently reported in self-management programmes for people with diabetes. However, systematic review of studies where blood pressure was reported, showed similar reductions in SBP (standard effect size of 0.2) [24]. The effect sizes observed are relatively moderate, about one-third the size of those of antihypertensive drugs [26].

The effects of intervention on BMI were modest in comparison to the results of behavioural modification trials. A lifestyle intervention offered to people with type 2 diabetes in the Look Ahead trial found that among patients with an average BMI of 35, at one year, the percentage loss in body weight was 8% higher in the intervention group [27] (which is equivalent to a difference of approx 2.7 in BMI for their average 1.76 m individual). In our study, we observed overall smaller reductions, although with effects of this magnitude among those with morbid obesity (BMI ≥ 40). The difference in BMI between Birmingham Own Health^® ^and control participants in this category was equivalent to about 7% of initial body weight at follow up. This is likely to lead to significant health benefits in terms of glucose control and reduction in risk of cardiovascular events [28].

### Strengths and limitations

Although not randomised, this study used a novel approach for comparing the intervention group with matched controls from the general population in a primary care setting. The approach for identifying controls from GPRD is relatively unique, and the large number of controls from a wide population base increases the power of the study. The benefit of having a control group is evident from a comparison of mean change in clinical measures from baseline to follow-up, with the adjusted mean difference between groups. Using pre/post intervention changes only shows generally much larger effect sizes, which are partly due to regression to the mean. The inclusion of a control group provides a more realistic estimate of effect size, which is slightly attenuated. The Birmingham Own Health^® ^intervention is novel in targeting people from the most deprived communities, who are traditionally hard to reach. A recent analysis of the impact of the Quality Outcomes Framework indicated that it has led to improved quality of care in more deprived practices [29], but may not have resulted in improved health in those most deprived or indeed reduced inequalities in health. The Birmingham Own Health intervention appeared to be effective in the most deprived areas and therefore has potential to be of benefit in improving the health of those least well off. A caveat is that the measure of deprivation is at area level rather than individual level, so this effect cannot necessarily be assumed. The use of telephone intervention overcomes many of the barriers reported for non-participation in self-management programmes, namely travel, inconvenience and timing of sessions [30]. Furthermore, the range of clinical indicator outcomes measured is wider than those in some previous studies.

On the other hand, lack of randomisation is an important potential source of selection bias. We minimised this by matching controls to cases as closely as possible in relation to broad inclusion and exclusion criteria, to baseline test values and to the dates when these were measured. Furthermore we adjusted for differences in the analyses, thus minimising further the effect of any bias. Nevertheless we were only able to match on limited measures and it is possible that cases and controls differed in other ways that we have not captured. In fact cases and controls did differ in relation to comorbidities, but this was difficult to control because of poor recording of conditions in both cases and controls. Similarly recording errors for other confounding factors including medications and smoking make adjustment unreliable. The "opt-out" system may also have meant that the most motivated patients joined the intervention, potentially overestimating the effects of the intervention.

The way in which follow up data was obtained differed between cases and controls. For GPRD controls, these measures were taken from GP record data. However Birmingham Own Health^® ^participants reported the measures themselves, and social acceptability bias is a possibility. Although a number of outcome measures were included, some important measures, such as change in smoking status or exercise levels were not recorded. This limits the measure of effectiveness and the wider potential effects of the intervention. It is also important to consider that "usual care" in the control group is not uniform, and may have also changed over time. Thus the effectiveness of the Birmingham Own Health^® ^intervention is not in comparison to no intervention. By selecting controls with measures taken within +/- 90 days of the intervention participants, the effects of change in background levels of "usual care" was avoided.

## Conclusions

Despite the limitations of the study design, the results are consistent with the Birmingham Own Health^® ^telephone care management intervention being effective in reducing HbA1c levels, blood pressure and BMI in people with diabetes, and importantly, to a degree comparable with randomised controlled trials of similar interventions. The effects appear to be greater in patients with poorer baseline levels and the intervention appears effective in the most deprived populations. The levels of change observed are associated with reductions in expected all-cause and cardiovascular mortality, based on observational studies. Although the cost-effectiveness of this intervention is not known, economic evaluation of another telephone self-management intervention for people with diabetes found that the cost per QALY was favourable compared to other established diabetes prevention and treatment interventions [31]. This novel methodology appears to offer a relatively cheap alternative to RCTs.

## Competing interests

All authors have completed the manuscript submission forms and declare that they have received financial support from Pfizer Health Solutions and Birmingham East and North PCT for the submitted work. However, the interpretation and conclusions contained in this study are those of the authors alone.

## Authors' contributions

PA and RJ designed the protocol and obtained the GPRD data. RL extracted and prepared the data. RJ & RL undertook the analyses with advice from PA. RJ and PA co-wrote the paper. All authors take responsibility for the integrity of the data and accuracy of the data analysis. All authors have read and approved the final manuscript.

## Pre-publication history

The pre-publication history for this paper can be accessed here:

http://www.biomedcentral.com/1471-2458/11/707/prepub
